# Protective Properties of Silane Composite Coatings Modified with Poly(3,4-ethylenedioxythiophene) with Heteropolyacid on X20Cr13 and 41Cr4 Steel

**DOI:** 10.3390/ma17246177

**Published:** 2024-12-18

**Authors:** Aleksandra Kucharczyk-Kotlewska, Lidia Adamczyk, Krzysztof Miecznikowski, Agata Dudek

**Affiliations:** 1 Department of Materials Engineering, Faculty of Production Engineering and Materials Technology, Czestochowa University of Technology, Aleja Armii Krajowej 19, 42-200 Czestochowa, Poland; aleksandra.kucharczyk@pcz.pl (A.K.-K.); lidia.adamczyk@pcz.pl (L.A.); 2 Faculty of Chemistry, University of Warsaw, Ludwika Pasteura 1, 02-093 Warsaw, Poland; kmiecz@chem.uw.edu.pl

**Keywords:** vinyltrimethoxysilane (VTMS), poly(3,4-ethylenedioxythiophene) PEDOT, heteropolyacid, phosphomolybdic acid (PMo_12_)

## Abstract

This paper describes the methodology of the preparation and analyses of the structure and anticorrosion properties of silane coatings modified with poly(3,4-ethylenedioxythiophene) (PEDOT) with phosphododecamolybdic acid (PMo_12_). Protective coatings, consisting of vinyltrimethoxysilane (VTMS), PEDOT powder with PMo_12_ admixture (at different concentrations), and ethanol, were deposited on X20Cr13 and 41Cr4 steels by immersion. The physicochemical properties of these silane coatings (e.g., surface morphology, thickness, roughness, and adhesion to the substrate) were elucidated using a digital microscope, a Fourier transform infrared spectrophotometer with attenuated total reflectance, and various electrochemical diagnostic techniques. Protective properties were assessed in acidified sulfate solutions with and without chloride ions (pH 2). Experimental results have shown that this coating displayed the effective protection of steel against general and pitting corrosion, stabilized the corrosion potential in the passive state, and provided barrier protection.

## 1. Introduction

Stainless steel has been widely used in many industries, especially those where high-quality and durable materials are critical. In the construction industry in particular, it is valued for its modern, elegant appearance and its ability to be easily formed into a variety of shapes [1,2,3,4]. With its resistance to corrosion, mechanical damage, and extreme temperatures, stainless steel is an ideal material for the manufacture of components such as pipes, balustrades, columns, or lift cabins [5,6,7,8]. Despite its favorable properties, stainless steel, including steel with more than 10.5% chromium, can rust under adverse conditions. Any steel reacts with oxygen to form an oxide layer. In conventional steels, this process leads to corrosion as oxygen reacts with the iron atoms to form a rougher surface. In stainless steel, chromium reacts with oxygen to form a passive layer that protects the material from further corrosion reactions [9,10,11].

Advanced protective coating technologies are being developed to increase the protection of steel against corrosion [12,13]. One such technology is silane coatings, which contain both organic (methoxy or ethoxy) groups and inorganic silicon [14,15,16,17]. Silanes were initially used as adhesion promoters for glass substrates, but their use has expanded significantly to include corrosion protection for various metals such as aluminum, iron, and steel [18,19,20,21,22,23,24,25,26]. Silane technology became particularly important in the 1990s when alternatives to toxic chromium (VI) coatings were sought [27,28,29,30].

In addition to silanes, conductive polymers such as poly(3,4-ethylenedioxythiophene) (PEDOT) are beginning to play an increasingly important role in corrosion protection [31,32,33,34,35,36,37,38,39,40]. Due to their conductive properties, these polymers find applications in a wide range of fields, from electronics to sensors and catalysis [41,42,43,44,45,46,47]. By combining silane coatings with conductive polymers such as PEDOT, hybrid coatings can be created that significantly improve the corrosion protection of steel while providing additional functions such as electrical conductivity [48,49,50,51,52,53].

PEDOT (poly(3,4-ethylenedioxythiophene)) coatings can be obtained by chemical or electrochemical polymerization of the EDOT monomer in organic solvents such as H_2_SO_4_ [54,55,56,57]. A study [58] has shown that the use of the micellar agent BRIJ significantly improves the degree of cross-linking and structural properties of PEDOT coatings, resulting in better protective and functional performance.

A literature review [59,60,61,62,63] also suggests that phosphoric acid and heteropolyacids (HPAs) play a key role in the electropolymerization of thiophenes, leading to the formation of hybrid PEDOT coatings with polyoxometalates such as phosphomolybdate and phosphovolphramate. HPAs are distinguished by their strong acidic properties [64,65] and excellent proton conductivity under ambient conditions [66,67,68]. Their ability to rapidly and reversibly transfer electrons makes them of particular interest as potential electrocatalysts in redox reactions [69,70,71,72,73,74]. Such hybrid composite coatings, combining the properties of PEDOT and HPA, represent a promising solution for corrosion protection and functional materials in advanced technological applications [75,76,77,78,79,80,81].

The aim of this study was to develop a method for the deposition of anticorrosion coatings on X20Cr13 and 41Cr4 steels using the EDOT monomer with phosphododecamolybdic acid (H_3_PMo_12_O_40_) in a sol–gel solution containing VTMS silane. PEDOT was chosen for its high chemical stability, low toxicity, and ease of application, making it a suitable material for corrosion protection. In the present study, the proposed coatings displayed the effective protection of steel against general and pitting corrosion (presence of phosphododecamolybdic anions), stabilized the corrosion potential in the passive state (existence of conducting polymer—PEDOT), and provided anodic barrier protection. Furthermore, despite the extensive use of PEDOT in other areas, the use of VTMS/PEDOT/PMo_12_ coatings in the corrosion protection of X20Cr13 and 41Cr4 steels has not been reported in the literature.

## 2. Materials and Methods

### 2.1. Materials

The following reagents were used to prepare the modification solution: vinyltrimethoxysilane (VTMS 99%, Sigma Aldrich, St. Louis, MO, USA), anhydrous ethyl alcohol (EtOH, Sigma Aldrich, St. Louis, MO, USA), 3,4-ethylenedioxythiophene monomer (EDOT, Sigma Aldrich, St. Louis, MO, USA), and phosphododecamolybdic acid (PMo_12_, Fluka, Buchs, Switzerland). All reagents had a p.a. purity grade.

Table 1 illustrates the chemical composition of the alloy steels used in the experiments. Steel specimens with a diameter of 5 mm were placed in polymethylmethacrylate holders using epoxy resin. The working area of the specimens was 0.2 cm^2^. The specimens were mechanically polished on increasingly fine-grained sandpaper up to No. 2000 each time before conducting experiments, followed by washing with distilled water and ethanol. Before the application of the coating, each specimen was washed with acetone to degrease the surface.

### 2.2. Coating Preparation

To prepare the modifying solution, 4.84 mL of 3.16 mol dm^−3^ vinyltrimethoxysilane (VTMS) was used along with 2.16 mL of EtOH. The solution was placed on a magnetic stirrer and stirred for 2 days at 800 to 1000 rpm. A powder consisting of EDOT and PMo_12_ was then added. The procedure for obtaining PEDOT/PMo_12_ powder was described in a previous publication [60].

Different amounts of powder added to the silane solution were used in the experiment, i.e., 0.1 g, 0.15 g, 0.25 g, and 0.35 g. The solution was placed again on a magnetic stirrer for 24 h to obtain a gel consistency. The experiments were conducted at room temperature of 24 °C in laboratory settings.

VTMS/PEDOT/PMo_12_ coatings were applied to X20Cr13 and 41Cr4 steels by immersion for 20 min. The specimens were placed in a silica gel desiccator until the coatings dried completely (about 2 days).

### 2.3. Characteristics of Coatings

Microstructural examinations of coatings deposited on X20Cr13 and 41Cr4 steel were performed using a KEYENCE VHX—7000 digital microscope (Keyence, Mechelen, Belgium). The adhesion of VTMS/PEDOT/PMo_12_ coatings was tested using Scotch^TM^ tape (Scotch^TM^ Brand, St. Paul, MN, USA) with the pull-off method according to the ASTM D3359 standard [82].

The thickness of the coatings was measured using a Testan DT-20 AN 120 157 m (Anticorr, Gdańsk, Poland) with an integrated probe designed for ferromagnetic and non-ferromagnetic measurements. Corrosion resistance tests were carried out using a CH Instruments 706 measuring station (Austin, TX, USA). A three-electrode system was used: specimens of X20Cr13 and 41Cr4 steel without and with a coating: working electrode, counter-electrode (platinum wire), and reference electrode (saturated calomel electrode SCE). The properties of the anti-corrosion coatings were evaluated in two corrosion environments, 0.5 mol dm^−3^ Na_2_SO_4_ (pH = 2) and 0.5 mol dm^−3^ Na_2_SO_4_ + 0.5 mol dm^−3^ NaCl (pH = 2), using the Tafel Plot potentiodynamic technique, using potential scanning from −0.8 V to +1.6 V with a polarization rate of 10 mVs^−1^. The tests were conducted at room temperature.

## 3. Results and Discussion

**Figure 1:**
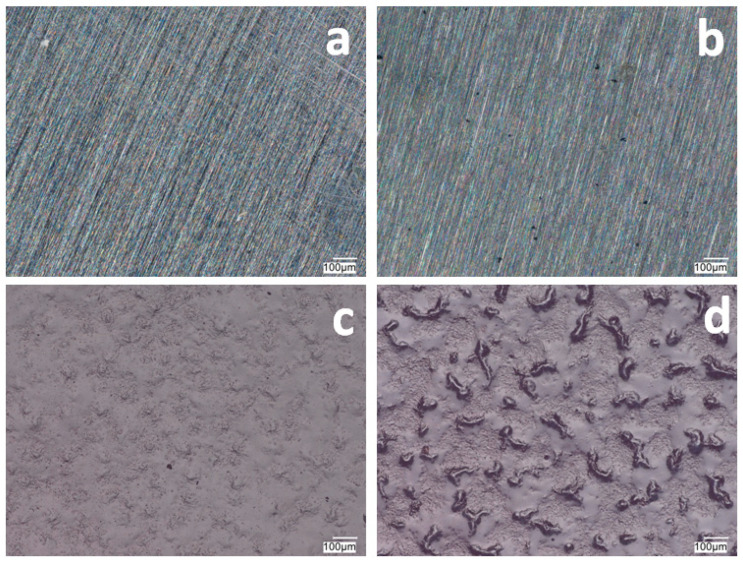
Surface of VTMS/PEDOT/PMo_12_ coatings deposited on X20Cr13 steel with VTMS concentration of 3.16 mol dm^−3^ and EDOT/PMo_12_ powder content: 0.1 g (**a**), 0.15 g (**b**), 0.25 g (**c**), and 0.35 g (**d**). KEYENCE VHX-7000 digital microscope, Magn. 200.

**Figure 2:**
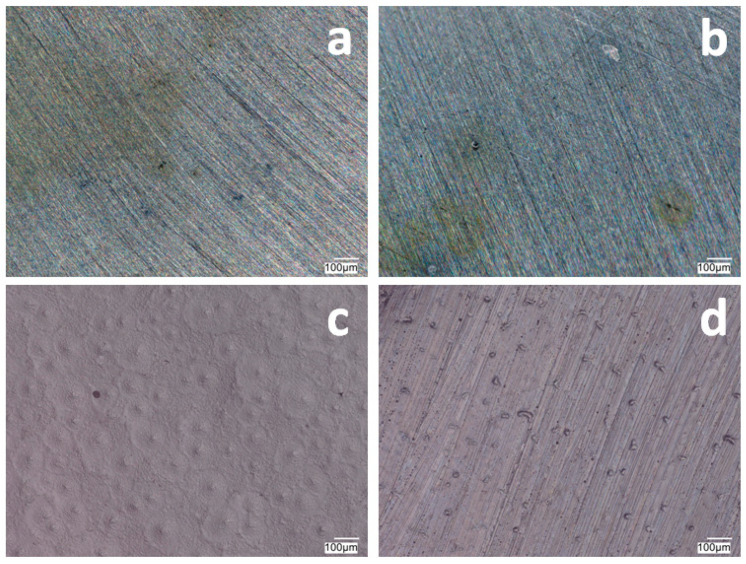
The surface of VTMS/PEDOT/PMo_12_ coatings deposited on 41Cr4 steel with VTMS concentration of 3.16 mol dm^−3^ and EDOT/PMo_12_ powder content: 0.1 g (**a**), 0.15 g (**b**), 0.25 g (**c**), and 0.35 g (**d**). KEYENCE VHX-7000 digital microscope, Magn. 200.

Figure 1 and Figure 2 show the morphology of the VTMS/PEDOT/PMo_12_ coatings deposited on the surface of X20Cr13 and 41Cr4 steels. The surface and morphology of VTMS/PEDOT/PMo_12_ coatings deposited on X20Cr13 and 41Cr4 steels, at different concentrations of EDOT/PMo_12_ powder, were strongly dependent on the content of the components used in the deposition process. At the lowest powder concentration, 0.1 g (1a, 2a) and 0.15 g (1b, 2b) of EDOT/PMo_12_, the VTMS/PEDOT/PMo_12_ coating on X20Cr13 steel showed a smooth and homogeneous surface. A low powder concentration leads to a thinner coating, which may exhibit poorer corrosion properties.

Increasing the EDOT/PMo_12_ powder content led to a more developed surface structure. As the concentration of EDOT/PMo_12_ increased, as with 0.25 g or 0.35 g, the coating became thicker and more opaque. The coatings exhibited a slightly rough structure, which increased the active surface area. The increase in powder concentration led to an increase in the thickness of the coating, which improved its ability to protect against external agents. The coating at 0.25 g or 0.35 g EDOT/PMo_12_ powder was characterized by a more complex morphology, with visible aggregates and pronounced surface irregularities. At this concentration, the coating can be expected to have better conductive properties due to the higher amount of polymer and heteropolyacid. However, higher roughness can also increase the risk of penetration by corrosive agents, which can limit the durability of the coating if adequate barrier properties are not provided.

### 3.1. Scotch Test

To confirm the results of the adhesion of the VTMS/PEDOT/PMo_12_ coating, an adhesion test was performed using Scotch^TM^ tape. The test was performed immediately after the deposition of VTMS/PEDOT/PMo_12_ coatings on the X20Cr13 and 41Cr4 steels. The tests conducted by the relevant standard showed that coatings containing 0.1 g and 0.15 g of EDOT/PMo12 powder demonstrated poor adhesion to the substrate. A material loss of approximately 30–40% of the coating was observed on X20Cr13 and 41Cr4 steels (classification 1B). In contrast, coatings with 0.25 g and 0.35 g of EDOT/PMo12 powder exhibited a material loss of less than 5% (classification 4B), indicating very good adhesion of the coatings to the substrate of both steels.

### 3.2. Thickness of the Coatings

**Figure 3:**
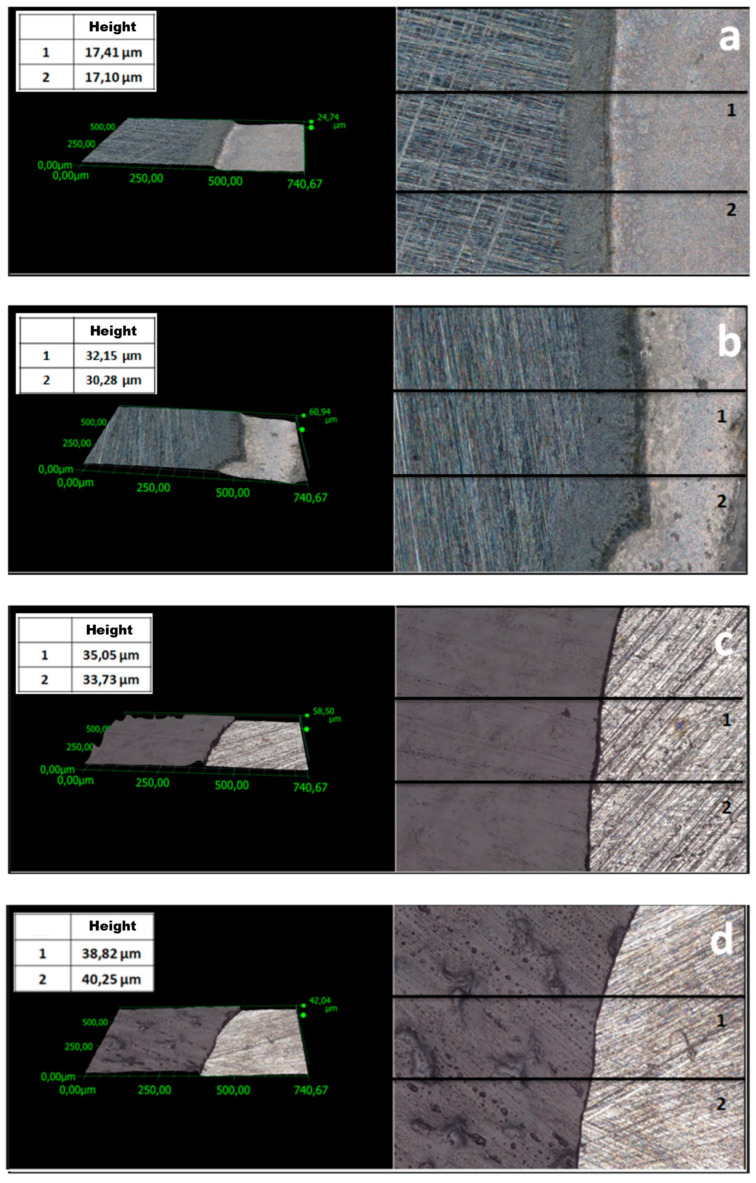
Surface profile of VTMS/PEDOT/PMo_12_ coatings deposited on X20Cr13 steel with different EDOT/PMo_12_ powder content: 0.1 g (**a**), 0.15 g (**b**), 0.25 g (**c**), 0.35 g (**d**).

**Figure 4:**
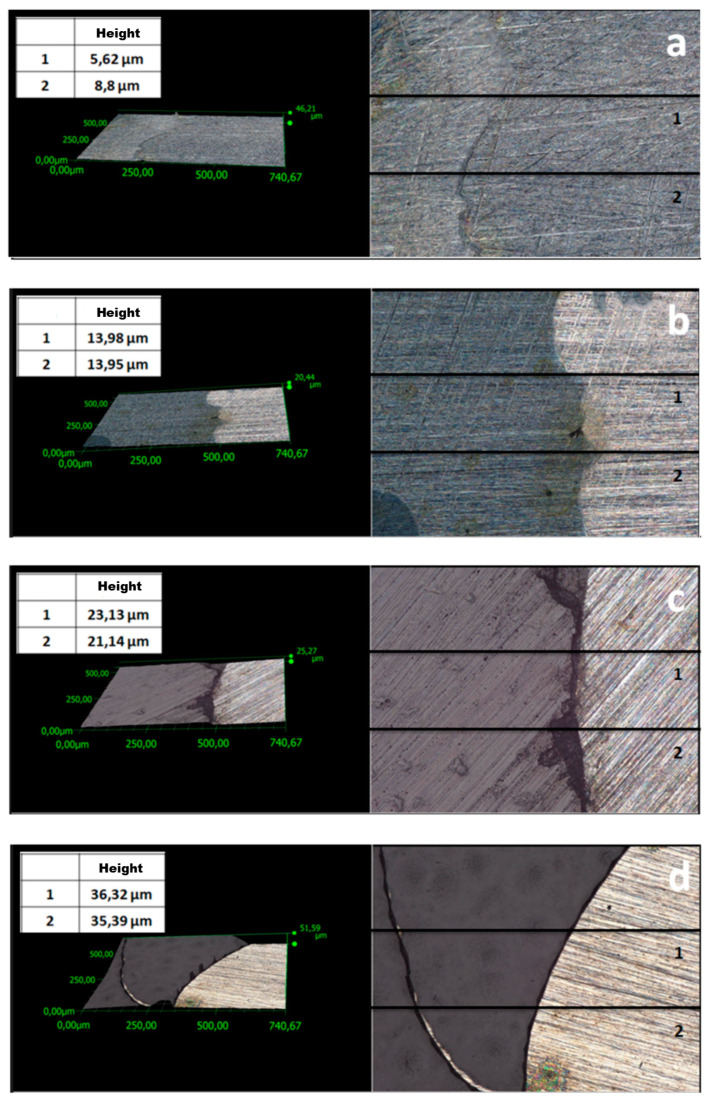
Surface profile of VTMS/PEDOT/PMo_12_ coatings deposited on 41Cr4 steel with different EDOT/PMo_12_ powder content: 0.1 g (**a**), 0.15 g (**b**), 0.25 g (**c**), 0.35 g (**d**).

One of the key parameters affecting the corrosion resistance of steel components is the thickness of protective coatings. In this study, this parameter was analyzed using two methods. Profiles were examined (Figure 3 and Figure 4) to evaluate the thickness of the coatings. The average thickness of VTMS/PEDOT/PMo_12_ coatings with variable EDOT/PMo_12_ powder content (measured at 2 locations on the specimen) is presented in Table 2. The recorded thickness was measured using a KEYENCE digital microscope for different EDOT/PMo_12_ powder content: 0.1 g (a), 0.15 g (b), 0.25 g (c), and 0.35 g (d).

To compare coating thickness, in addition to the method described above, thickness measurements were performed using a Testan meter. A series of 10 measurements (at different sites on the specimens) was performed. Table 2 shows the average thickness for the individual VTMS/PEDOT/PMo_12_ coatings. The results are consistent with the thickness evaluated using a digital microscope. Based on measurements using two instruments (digital microscope and thickness gauge), the mean coating thickness was determined (Table 2).

The differences in coating thickness between the methods were small, confirming the suitability of the methods used to measure coating thickness. Based on the thickness measurements, it can be concluded that as the amount of EDOT/PMo_12_ powder in the coating increased, the thickness of the coating also increased.

### 3.3. Analysis of 2D Surface Geometry: Roughness Parameter

The analysis of 2D surface geometry was carried out by measuring the Ra parameter (Table 3). Test results varied depending on the content of the EDOT/PMo_12_ powder used in the modifying solution. As the content of the EDOT/PMo_12_ powder in the modifying solution increased, the value of the Ra parameter for the tested VTMS/PEDOT/PMo_12_ coatings also increased. The more EDOT/PMo_12_ powder in the modifying solution, the rougher the coating structure.

The closest Ra values were observed for VTMS/PEDOT/PMo_12_ coatings with EDOT/PMo_12_ powder contents of 0.1 g and 0.15 g and those with 0.25 g and 0.35 g for X20Cr13 steel. For 41Cr4 steel, the VTMS/PEDOT/PMo_12_ coatings with EDOT/PMo_12_ powder contents of 0.15 g and 0.25 g had the most similar Ra values. The lowest surface roughness was recorded for the VTMS/PEDOT/PMo_12_ coating with an EDOT/PMo_12_ powder content of 0.1 g for 41Cr4 steel.

### 3.4. Analysis of Coating Composition

The characterization of coatings based on vinyltrimethoxysilane VTMS, poly(3,4-ethylenedioxythiophene) PEDOT, and phosphomolybdic acid H_3_[Mo_12_PO_40_]*12H_2_O deposited on steels (X20Cr13 and 41Cr4) was determined using the Fourier transform infrared spectrophotometer with attenuated total reflectance (QATR-FTIR) spectrophotometer method (Bruker, Bremen, Germany). A detailed description with drawings of the absorbance peaks for VTMS, PEDOT, and PMo_12_ coatings was presented in previous publications [47,50,57].

**Figure 5:**
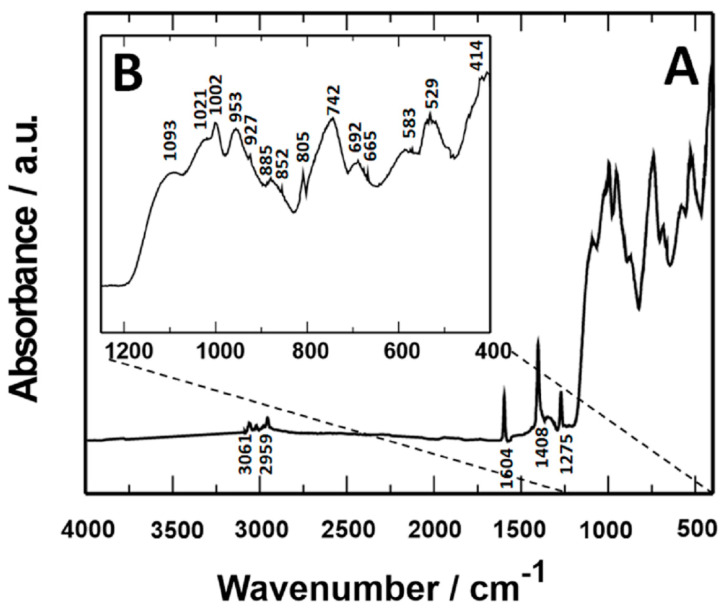
FTIR spectrum of VTMS/PEDOT/PMo_12_ coating with 0.25 g EDOT/PMo_12_ powder content, X20Cr13 steel substrate. (**A**) FTIR spectrum in the range 4000–400 cm^−1^, (**B**) FTIR spectrum in the range 1200–400 cm^−1^.

The VTMS/PEDOT/PMo_12_ coating spectrum cannot be considered a simple combination of VTMS, PEDOT, and PMo_12_ spectra. The characteristic bands of VTMS/PEDOT/PMo_12_ coatings have higher absorbance and wavelength shifts compared to VTMS, PEDOT, and PMo_12_ spectra. Due to the similar appearance (analogous course) of the other coatings, the FTIR spectrum for the VTMS/PEDOT/PMo_12_ coating with an EDOT/PMo_12_ powder content of 0.25 g (X20Cr13 steel) is shown in Figure 5.

Characteristic absorbance peaks observed for VTMS/PEDOT/PMo_12_ coatings in the 4000–400 cm^−1^ range are as follows:

- Absorbance bands observed at values of 3061 cm^−1^, 2959 cm^−1^, and 1275 cm^−1^ correspond to the asymmetric stretching and bending vibrations of the C-H bond belonging to the -Si-(OCH_3_) group;
- Further peaks were recorded at 1604 cm^−1^ and 1408 cm^−1^, which correspond to stretching vibrations of the C=C bond of the CH_2_=CH-group;
- 1002 cm^−1^, 885 cm^−1^, and 742 cm^−1^ bands correspond to Si-O-C vibration;
- A broad band observed at 1190–1000 cm^−1^ corresponds to asymmetric stretching vibrations of Si-O-Si bonds;
- A peak at 692 cm^−1^ corresponds to the Si-C bond;
- A peak at 927 cm^−1^ was attributed to asymmetric bending vibrations of the Si-OH bond;
- 1021 cm^−1^, 953 cm^−1^, 852 cm^−1^, and 805 cm^−1^—four characteristic bands of the PMo_12_ ion, which can be related to asymmetric stretching with edge oxygen, respectively: P-O, Mo=O, Mo-O_c_-Mo, and Mo-O_e_-Mo;
- 583 cm^−1^: the band attributed to the Keggin δ (O-P-O) structure;
- 529 cm^−1^ and 414 cm^−1^: sharp Si-O bond bands.

### 3.5. Corrosion Testing

**Figure 6:**
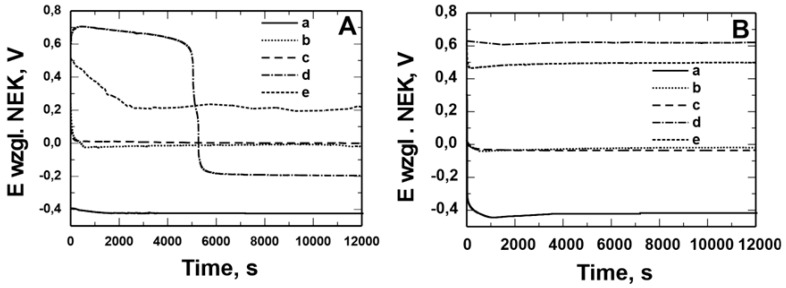
Measurement of the open potential of OCP from the time of exposure in solution: 0.5 mol dm^−3^ Na_2_SO_4_ pH = 2 (**A**) and 0.5 mol dm^−3^ Na_2_SO_4_ + 0.5 mol dm^−3^ NaCl pH = 2 (**B**) for X20Cr13 steel without (**a**) and with VTMS/PEDOT/PMo_12_ coatings with EDOT/PMo_12_ powder content: 0.1 g (**b**), 0.15 g (**c**), 0.25 g (**d**), 0.35 g (**e**).

**Figure 7:**
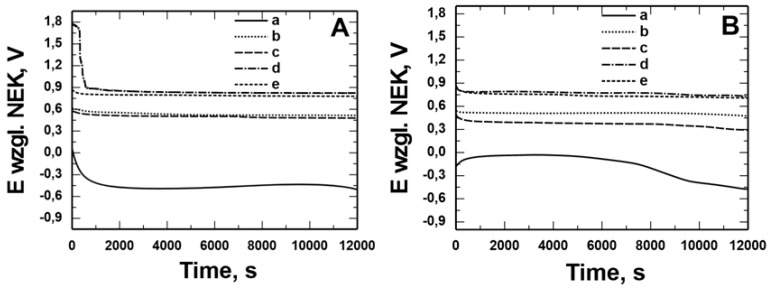
Measurement of the open potential of OCP from the time of exposure in solution: 0.5 mol dm^−3^ Na_2_SO_4_ pH = 2 (**A**) and 0.5 mol dm^−3^ Na_2_SO_4_ + 0.5 mol dm^−3^ NaCl pH = 2 (**B**) for 41Cr4 steel without (**a**) and with VTMS/PEDOT/PMo_12_ coatings with EDOT/PMo_12_ powder content: 0.1 g (**b**), 0.15 g (**c**), 0.25 g (**d**), 0.35 g (**e**).

Figure 6 and Figure 7 show the open circuit potential (OCP) measurements recorded in solution: 0.5 mol dm^−3^ Na_2_SO_4_ pH = 2 and 0.5 mol dm^−3^ Na_2_SO_4_ + 0.5 mol dm^−3^ NaCl pH = 2 for X20Cr13 (Figure 6) and 41Cr4 (Figure 7) steels without (a) and with VTMS/PEDOT/PMo_12_ coating with EDOT/PMo_12_ powder content: 0.1 (b), 0.15 (c), 0.25 (d), and 0.35 (e) g. Uncoated X20Cr13 steel immediately after immersion in corrosive solutions showed potential of approximately −0.4 V. At longer exposure times, the corrosion potential value for the steel was −0.5 V. In contrast, 41Cr4 steel without coating immediately after immersion in corrosive solutions showed potential of −0.3 V to −0.6 V. For X20Cr13 and 41Cr4 steel with VTMS/PEDOT/PMo_12_ coatings, the potential remained in the range of 0.9 to −0.6 V, which was due to the different EDOT/PMo_12_ powder content (Table 4) in the sol–gel solution.

**Figure 8:**
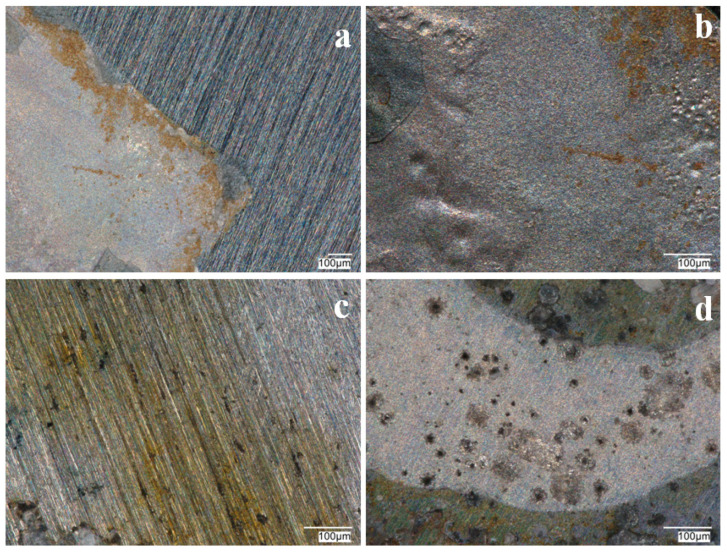
Photographs of VTMS/PEDOT/PMo_12_ coatings after OCP testing in a solution of 0.5 mol dm^−3^ Na_2_SO_4_ + 0.5 mol dm^−3^ NaCl pH = 2. Content of EDOT/PMo_12_ powder: 0.1 (**a**,**c**), 0.15 (**b**,**d**), substrate: X20Cr13 steel (**a**,**b**), 41Cr4 steel (**c**,**d**). KEYENCE VHX-7000 digital microscope, Magn. 200.

Figure 8 shows microscopic observations made after measuring OCP in a sulfate solution with the addition of Cl^−^ ions. VTMS/PEDOT/PMo_12_ coatings with EDOT/PMo_12_ powder contents of 0.1 g and 0.15 g showed local corrosion effects (pitting), indicating ineffective corrosion protection. No pitting corrosion was observed for the coating with the higher powder content in the sol–gel.

To determine the most effective effect of EDOT/PMo_12_ powder content on the corrosion properties of the VTMS/PEDOT/PMo_12_ coatings on X20Cr13 and 41Cr4 steels, their ability to inhibit general and pitting corrosion was evaluated using potentiodynamic curves. Potentiodynamic measurements were performed in the log(i) = f(E) configuration and the curves were recorded at a polarization rate of 10 mVs^−1^. This scan rate was sufficient to register Faradaic processes on the electrode. The total duration of a single measurement was very short, lasting 4 min.

**Figure 9:**
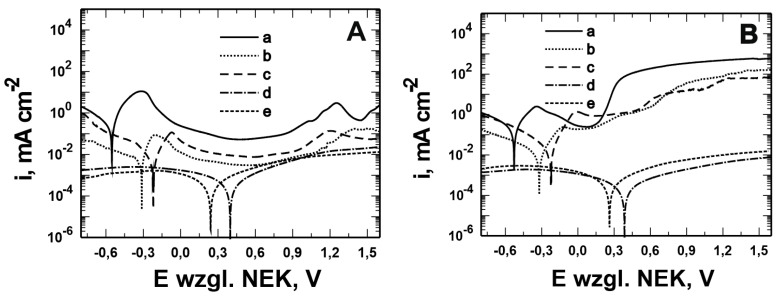
Potentiodynamic polarization curves recorded in the solution: 0.5 mol dm^−3^ Na_2_SO_4_ pH = 2 (**A**) and 0.5 mol dm^−3^ Na_2_SO_4_ + 0.5 mol dm^−3^ NaCl pH = 2 (**B**) for X20Cr13 steel without (**a**) and with VTMS/PEDOT/PMo_12_ coatings with powder content EDOT/PMo_12_ in solution: 0.1 (**b**), 0.15 (**c**), 0.25 (**d**), and 0.35 (**e**) g. Polarization rate 10 mVs^−1^, solutions in contact with air.

**Figure 10:**
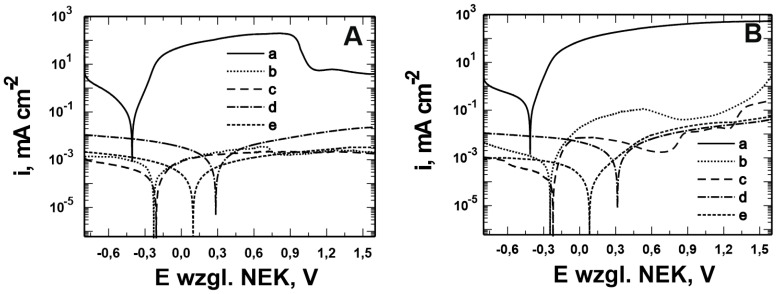
Potentiodynamic polarization curves recorded in the solution: 0.5 mol dm^−3^ Na_2_SO_4_ pH = 2 (**A**) and 0.5 mol dm^−3^ Na_2_SO_4_ + 0.5 mol dm^−3^ NaCl pH = 2 (**B**) for 41Cr4 steel without (**a**) and with VTMS/PEDOT/PMo_12_ coatings with powder content EDOT/PMo_12_ in solution: 0.1 (**b**), 0.15 (**c**), 0.25 (**d**), and 0.35 (**e**) g. Polarization rate 10 mVs^−1^, solutions in contact with air.

The experiment was conducted in two corrosion solutions: 0.5 mol dm^−3^ Na_2_SO_4_ pH = 2—general corrosion (Figure 9A and Figure 10A) and 0.5 mol dm^−3^ Na_2_SO_4_ + 0.5 mol dm^−3^ NaCl pH = 2—pitting corrosion (Figure 9B and Figure 10B). The potential range was −0.8 ÷ 1.6 V for X20Cr13 and 41Cr4 steel, uncoated and coated, respectively. As shown in Figure 9A and Figure 10A, the VTMS/PEDOT/PMo_12_ coatings obtained in the study inhibited cathodic and anodic processes. There was a shift in the corrosion potential of the following aspects:

- X20Cr13 by approximately 0.9 V compared to uncoated steel (VTMS/PEDOT/PMo_12_ coating with 0.25 g of EDOT/PMo_12_ powder);
- 41Cr4 by approximately 0.7 V compared to uncoated steel (VTMS/PEDOT/PMo_12_ coating with 0.25 g of EDOT/PMo_12_ powder).

Anodic current densities for both steels with VTMS/PEDOT/PMo_12_ coatings in the passive range were 1 to 3 times lower than for uncoated steel.

Table 5 presents the data obtained from the Tafel plot and the polarization resistance calculated for X20Cr13 steel.

For the 41Cr4 steel, the calculated polarization resistance values are as follows—for general corrosion, the following values were obtained for coating powders: 0.1 g–15.5007 Ωcm^2^, 0.15 g–26.3226 Ωcm^2^, 0.25 g–39.4664 Ωcm^2^, and 0.35 g–84.7618 Ωcm^2^; and for pitting corrosion: 0.1 g–9.7513 Ωcm^2^, 0.15 g–15.731 Ωcm^2^, 0.25 g–27.1054 Ωcm^2^, and 0.35 g–99.615 Ωcm^2^. The data obtained show that the polarization resistance was highest for both steels coated with 0.35 g of powder, indicating good corrosion protection.

To evaluate the ability of the obtained coatings to inhibit pitting corrosion, potentiodynamic curves were obtained for a solution of 0.5 mol dm^−3^ Na_2_SO_4_ + 0.5 mol dm^−3^ NaCl pH = 2 (Figure 9B and Figure 10B). The corrosion potential of X20Cr13 steel for all coatings shifted by about 0.2–0.9 V toward positive values compared to the corrosion potential recorded for uncoated steel (E_corr_ = −0.527 V). For 41Cr4 steel, the corrosion potential shifted toward positive values by approximately 0.2–0.7 V compared to the corrosion potential recorded for uncoated steel (E_corr_ = −0.45 V). Lower cathodic and anodic current density values were observed for both steels compared to uncoated steels. The polarization curves show that the pitting nucleation potential (E_pit_) was 0.12 V for uncoated X20Cr13 steel, whereas for the steel coated with VTMS/PEDOT/PMo_12_ with EDOT/PMo_12_ powder content, it was: 0.3 V for 0.1 g and 0.75 V for 0.15 g. For 41Cr4 steel without coating, no pitting nucleation potential (E_pit_) was observed, while for VTMS/PEDOT/PMo_12_ coating, it was 0.6 V for 0.1 g and 0.9 V for 0.15 g. For the VTMS/PEDOT/PMo_12_ coating with 0.25 g and 0.35 g of EDOT/PMo_12_ powder, no pitting nucleation potential was observed.

**Figure 11:**
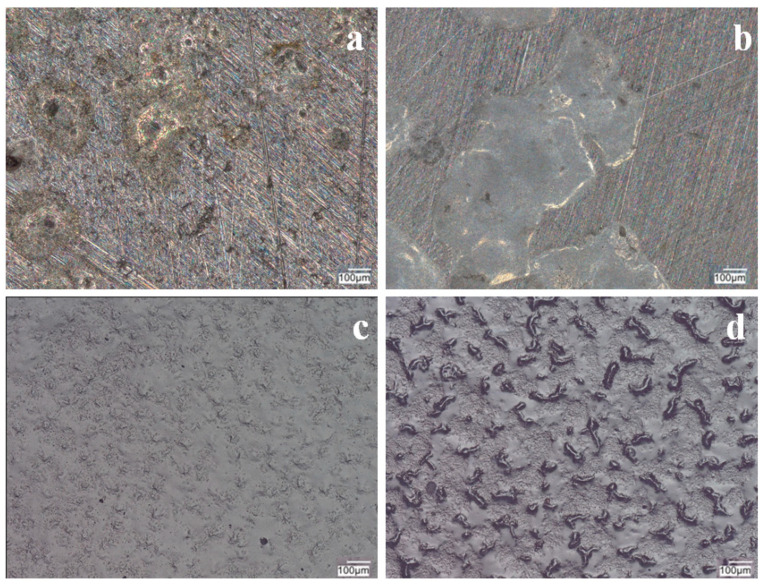
Photographs of VTMS/PEDOT/PMo_12_ coatings after potentiodynamic testing in a corrosion solution of 0.5 mol dm^−3^ Na_2_SO_4_ + 0.5 mol dm^−3^ NaCl pH = 2. Content of EDOT/PMo_12_ powder: 0.1 (**a**), 0.15 (**b**), 0.25 (**c**), and 0.35 (**d**) g, substrate X20Cr13 steel. KEYENCE VHX-7000 digital microscope, Magn. 200.

**Figure 12:**
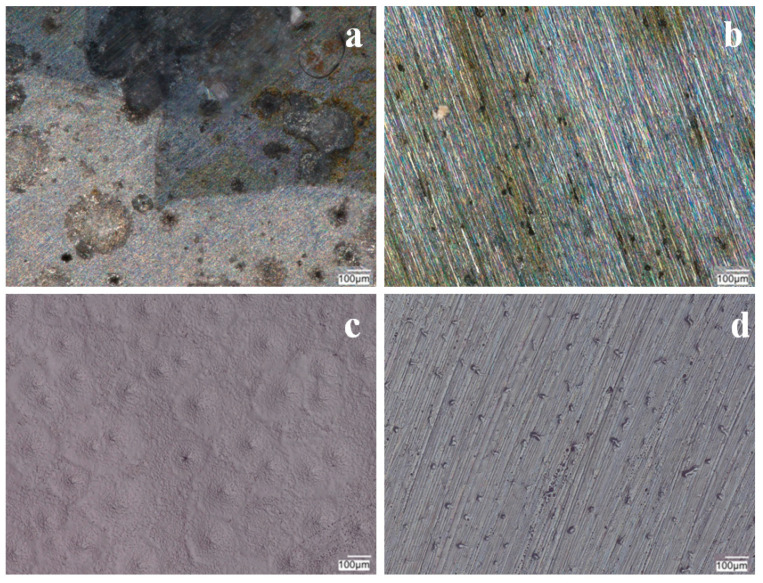
Photographs of VTMS/PEDOT/PMo_12_ coatings after potentiodynamic testing in a corrosion solution of 0.5 mol dm^−3^ Na_2_SO_4_ + 0.5 mol dm^−3^ NaCl pH = 2. Content of EDOT/PMo_12_ powder: 0.1 (**a**), 0.15 (**b**), 0.25 (**c**), and 0.35 (**d**) g, substrate 41Cr4 steel. KEYENCE VHX-7000 digital microscope, Magn. 200.

The silane coating with a conductive polymer and Keggin-type acid (heteropolyacid) effectively inhibited the access of aggressive anions to the steel substrate, thereby protecting the substrate from pitting corrosion. Microscopic observations made after the measurement showed no local corrosion effects under the VTMS/PEDOT/PMo_12_ coating for EDOT/PMo_12_ powder contents of 0.25 and 0.35 mol dm^−3^ for both steels (Figure 11 and Figure 12). The coating with 0.1 and 0.15 g of EDOT/PMo_12_ powder showed local pitting corrosion sites (Figure 10 and Figure 11a,b). The high content of EDOT/PMo_12_ powder in the coating affected the higher density of the modification solution, resulting in a thicker coating and increased roughness in the coating structure.

**Figure 13:**
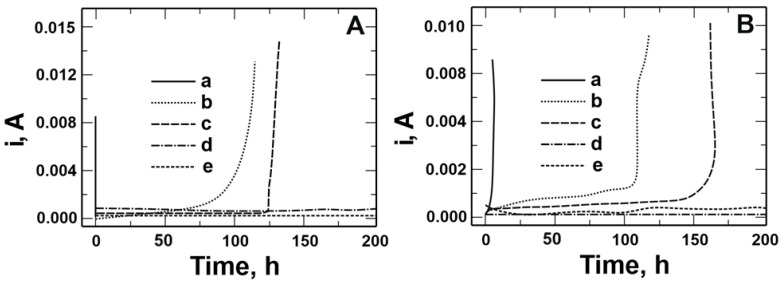
Chronoamperometric curves recorded in a solution of 0.5 mol dm^−3^ Na_2_SO_4_ + 0.5 mol dm^−3^ NaCl pH = 2 for steel: X20Cr13 (**A**) and 41Cr4 (**B**) without (**a**) and with VTMS/PEDOT/PMo_12_ coatings with EDOT/PMo_12_ powder content: 0.1 (**b**), 0.15 (**c**), 0.25 (**d**), and 0.35 (**e**) g.

A chronoamperometric method was used to verify the resistance to pitting corrosion of coatings deposited on steels (Figure 13A,B). In this method, changes in current density are recorded versus time when a constant potential is applied to the working electrode. Pitting nucleation can be inferred from the chronoamperometric curves. To determine the stability of the applied coatings, the time of the specimens remaining in the corrosive solution containing chloride ions and current density at the set potential were compared. Chronoamperometric curves were recorded in a solution of 0.5 mol dm^−3^ Na_2_SO_4_ + 0.5 mol dm^−3^ NaCl pH = 2 at a potential of 0.1 V for X20Cr13 steel and 0.5 V for 41Cr4 steel. As can be observed, the initiation of pitting formation on both steels occurred within a few seconds, followed by a rapid increase in current density. For VTMS/PEDOT/PMo_12_ coatings with EDOT/PMo_12_ powder contents of 0.25 and 0.35 g, the highest corrosion resistance was obtained for both steels. No increase in current density was observed for the above coatings during the 200 h measurement. An increase in current density was recorded over the time interval as follows:

- 50 to 100 h for VTMS/PEDOT/PMo_12_ coating with 0.1 g of EDOT/PMo_12_ powder for X20Cr13 and 41Cr4 steels;
- 120 to 125 h for VTMS/PEDOT/PMo_12_ coating with 0.15 g of EDOT/PMo_12_ powder for X20Cr13 steel;
- 125 to 160 h for VTMS/PEDOT/PMo_12_ coating with 0.15 g of EDOT/PMo_12_ powder for 41Cr4 steel.

The best ability to block the transport of chloride ions responsible for pitting corrosion was demonstrated by VTMS/PEDOT/PMo_12_ coatings with 0.25 and 0.35 g of EDOT/PMo_12_ powder (200 h).

## 4. Conclusions

In the present study, anticorrosion coatings based on vinyltrimethoxysilane (VTMS), poly(3,4-ethylenedioxythiophene) (PEDOT), and phosphododecamolybdic acid (PMo_12_) obtained on X20Cr13 and 41Cr4 steel by the sol–gel method were shown to be highly effective in terms of corrosion protection. The use of the immersion method allowed for coatings with high adhesion to be obtained, especially for specimens containing 0.25 g and 0.35 g of EDOT/PMo_12_ powder, which showed the best protective properties. These coatings were homogeneous, without cracks or defects, and their thickness depended on the amount of modifier powder added. The results of the surface morphology tests showed a low degree of roughness, an important factor in favor of corrosion resistance, especially for the specimens with 0.1 g of EDOT/PMo_12_ in the VTMS/PEDOT/PMo_12_ coating, where the lowest roughness (Ra) was recorded.

FTIR analysis confirmed the presence of significant chemical bonds, such as C-H, C=C, Si-O-C, Si-O-Si, P-O, and Mo=O, which are indicative of proper crosslinking of the coatings. Corrosion resistance tests were carried out in sulfate solutions, both without and with Cl- ions acidified to pH 2. These results indicated that the coatings effectively protected the steel against general and pitting corrosion, stabilized the corrosion potential in the passive state, and provided anodic barrier protection. Particularly effective were coatings with 0.25 g and 0.35 g of EDOT/PMo_12_ powder, which showed no signs of pitting corrosion even in the presence of chloride ions. OCP and chronoamperometric test results further confirmed the high resistance of these coatings to various forms of corrosion.

Based on the test results, it can be concluded that the proposed VTMS/PEDOT/PMo_12_ coatings significantly increase the corrosion resistance of X20Cr13 and 41Cr4 steels. Their protective properties, homogeneity, and stability in aggressive environments indicate their potential use in various industries where high corrosion resistance is crucial.

The coating described in this article can be used as a protective barrier in painting systems designed to protect steel structures. Due to its chemical stability and insolubility, it can provide effective protection against harmful external factors such as moisture and aggressive chemicals that can lead to steel corrosion.

The proposed protective coating, as demonstrated by the studies, exhibits high corrosion resistance, which can significantly extend the service life of steel structures, minimizing the need for frequent maintenance and repairs.

Thanks to its protective function, this coating could also serve as a foundation for the development of new material technologies capable of ensuring even better durability and efficiency, including in more demanding industrial conditions, such as environmental protection systems or catalytic processes in the chemical industry.

As part of future work, we plan to conduct further research on the use of other heteropolyacid structures, particularly those with much higher charge, such as Dawson-type heteropolyacids, as well as cesium salts of vanadium-substituted polyoxometalates, which are more stable and insoluble. These studies may contribute to the development of more resistant and efficient materials with improved catalytic and structural properties.

Additionally, compared to our previous studies [83] on silane coatings, it has been shown that the addition of EDOT/PMo12 powder to silane significantly improves the corrosion protection of the substrate. Furthermore, the results presented in the work [63] also show that the coating composed of EDOT/PMo_12_ exhibits lower corrosion resistance compared to the coating proposed in the current article. These facts unequivocally confirm the correctness of the research direction chosen by the authors, especially regarding the modification of the coating structure to improve the corrosion resistance of the substrate.

## Figures and Tables

**Table 1:** Chemical composition of the alloy steels used in the experiments.

	C %	Cr%	Si%	Ni%	Mn%	V%	P%	S%	Ti%	Mo%	W%
X20Cr13	0.17	12.6	0.34	0.25	0.30	0.04	0.024	<0.005	-	-	-
41Cr4	0.36–0.45	0.80–1.20	0.17–0.37	max 0.30	0.50–0.90	max 0.05	max 0.035	max 0.035	max 0.05	max 0.10	max 0.20

**Table 2:** Results of measurements of coating thickness using two instruments.

Instruments	Mean Thickness of VTMS/PEDOT/PMo_12_ Coating with Different EDOT/PMo_12_ Powder Content							
	0.1 g		0.15 g		0.25 g		0.35 g	
	Steel X20Cr13	Steel 41Cr4	Steel X20Cr13	Steel 41Cr4	Steel X20Cr13	Steel 41Cr4	Steel X20Cr13	Steel 41Cr4
KEYENCE VHX-7000 microscope	17.26 µm	7.24 µm	31.22 µm	13.97 µm	34.39 µm	22.14 µm	39.54 µm	35.86 µm
Testan DT-20 AN 120 157	16.58 µm	5.11 µm	29.59 µm	10.74 µm	32.81 µm	20.35 µm	37.27 µm	32.71 µm
Mean thickness (digital microscope and thickness gauge)	16.92 µm	6.16 µm	30.41 µm	12.36 µm	33.6 µm	21.25 µm	38.39 µm	34.29 µm

**Table 3:** Two-dimensional surface geometry analysis of VTMS/PEDOT/PMo_12_ coatings deposited on X20Cr13 and 41Cr4 steels: Ra parameter.

	2D Surface Roughness: Ra [µm]	
VTMS/PEDOT/PMo_12_ coating	X20Cr13 steel	41Cr4 steel
0.1 g of EDOT/PMo_12_ powder	3.66	1.83
0.15 g of EDOT/PMo_12_ powder	3.9	5.62
0.25 g of EDOT/PMo_12_ powder	5.24	6.97
0.35 g of EDOT/PMo_12_ powder	6.88	8.5

**Table 4:** Results of measurements of open circuit potential (OCP) for different times of exposure in corrosive solutions.

Corrosion Solution	X20Cr13 Steel		41Cr4 Steel	
	EDOT/PMo_12_ [g]	Potential [V]	EDOT/PMo_12_ [g]	Potential [V]
general corrosion 0.5 mol dm^−3^ Na_2_SO_4_ ph = 2	0.1	ca. −0.15 V	0.1	ca. 0.6 V
	0.15	ca. 0.0 V	0.15	ca. 0.57 V
	0.25	initially at 0.7 V, rapidly decreasing to −0.2 V after 5500 s	0.25	falls sharply to 0.9 V for the first 1000 s and remains at this level for a further 11,000 s
	0.35	for about 2500 s decreases from 0.5 V to 0.2 V and remains at 0.2 V for another 9500 s	0.35	ca. 0.85 V
pitting corrosion 0.5 mol dm^−3^ Na_2_SO_4_ + 0.5 mol dm^−3^ NaCl pH = 2	0.1	ca. −0.1 V	0.1	0.55 V
	0.15	ca. −0.08 V	0.15	0.5 V to 0.3 V
	0.25	ca. 0.6 V	0.25	ca. 0.87 V
	0.35	ca. 0.5 V	0.35	ca 0.87 V

**Table 5:** Data obtained from the Tafel plot and polarization resistance calculated for X20Cr13 steel.

Steel X20Cr13 VTMS/PEDOT/PMo_12_	Potential Corrosive E [V]		Current Density Corrosive i [mAcm^−2^]		Polarization Resistance tgα [Ω*cm^2^]	
	General Corrosion	Pitting Corrosion	General Corrosion	Pitting Corrosion	General Corrosion	Pitting Corrosion
0.1 g	−0.313	−0.319	8.08 × 10^−5^	9.765 × 10^−5^	0.50168	0.66663
0.15 g	−0.220	−0.219	6.705 × 10^−5^	4.8155 × 10^−4^	1.05892	0.26357
0.25 g	0.398	0.415	9.115 × 10^−8^	9.725 × 10^−5^	36.55406	61.10981
0.35 g	0.242	0.244	6.78 × 10^−6^	9.825 × 10^−5^	43.72182	85.44327

## Data Availability

The original contributions presented in this study are included in the article. Further inquiries can be directed to the corresponding author.
